# Impact of yoga way of life on organizational performance

**DOI:** 10.4103/0973-6131.72631

**Published:** 2010

**Authors:** Hasmukh Adhia, HR Nagendra, B Mahadevan

**Affiliations:** Government of Gujarat, Gulbai Tekra, Ahmedabad - 380 006, India; 1 Swami Vivekanand Yoga Anusandhan Samsthan, Kempegowda Nagar, Bangalore - 19, India; 2 Indian Institute of Management, Bannerghatta Road, Bangalore 560 076, India

**Keywords:** Yoga way of life, job satisfaction, job involvement, goal orientation, organizational citizenship behavior, affective organizational commitment

## Abstract

**Background:**

Organizational performance can be attributed to a number of factors. However, there are certain organizational factors, the presence or absence of which can determine the success or failure of the organization. There are different ways in which organizations try to improve their performance by working on such factors. In the research presented in this article, an attempt is made to find out whether adoption of the Yoga Way of Life by managers can have a positive impact on such organizational performance indicators.

**Aims:**

To measure effect of yoga way of life on five different indicators through an empirical study.

**Materials and Methods:**

The five indicators are job satisfaction, job involvement, goal orientation, affective organizational commitment and organizational citizenship behavior.

**Statistics Analysis:**

Pre- and post-data was measured using self-reported questionnaire. Independent T-test (Paired) and Pearson’s correlation test were conducted using SPSS.

**Results and Conclusion:**

The results of the study show that Yoga has a significant positive impact on four out of five of these indicators. Only job involvement does not show significant improvement. The construct used for measuring job involvement had a Chronbach alpha of 0.613, which is an indicator of moderate reliability, which could be the main reason for not getting positive result.

## INTRODUCTION

The globalization of the industrial world makes it imperative for organizations to put special emphasis on organizational innovation, flexibility, productivity, and responsiveness for changing the external conditions of their performance. Organizational performance can be measured in terms of different criteria for different organizations, and it depends to a great extent on the goals of an organization. However, one way of comparing organizations with different goals is to identify surrogate indicators of performance. In this article we have utilized the past studies and relevant literature to identify five organizational factors that can be used as alibis to assess the performance of an organization from the view point of the set objectives. These factors are common to most organizations, and therefore, can be used to make comparisons between companies or groups.

Today, there is considerable interest among the management practitioners and researchers with regard to the role and benefits of introducing spirituality at the workplace. The Harvard Business School study, drawn over a period of 11 years, showed a marked relation between the strength of the organizations’ corporate culture and its profitability.[1] Lloyd[2] maintains that organizations high in workplace spirituality outperform those without it by 86%. Taking a cue from such other studies, we have been motivated to introduce the concept of the ‘Yoga way of life’. We have analyzed the possible impact it can have on such organizational factors, and have utilized the empirical study and literature to make our inferences.

Yoga is generally perceived to be a way of keeping oneself healthy and happy. However, if one truly understands the concept of yoga as a complete way of life, one can clearly see its benefit for changing the paradigms of its practitioners. Such a change in the psycho-motivation of people is useful at the organizational level also. However, so far, very few empirical studies have been undertaken to establish such a link. The main contribution of this article is to fill this gap. Using a controlled scientific experimentation of employees in a manufacturing unit, we provide an empirical assessment of the impact of the yoga way of life on positive organizational factors.

We pose the question, “Can adoption of the yoga way of life make a positive impact on the factors which are responsible for the performance of organizations? If so, can we empirically observe this phenomenon and provide relevant literature support to explain this?” To the best of our knowledge, there is no empirical research available so far, to answer these questions. We study these issues in this article using an empirical study conducted in a manufacturing unit involving 84 executives.

We show that adoption of the yoga way of life can bring about better job satisfaction, affective organizational commitment, organizational citizenship behavior, and goal orientation of managers. These factors indeed contribute to the performance of the organization as we have argued in the article. Through a rigorous literature review and understanding of the science of Yoga, as given in our scriptures, we also provide an explanation of how this happens. We also motivate the HR managers in organizations to explore ways of implementing the yoga way of life, as it promises to address the issue of organizational climate at a fundamental level.

The rest of the article is organized as follows. In the next section we provide a review of the literature to introduce various factors contributing to the performance of an organization and the manner in which these contribute. On the basis of this, we identify the variables for our study. In Section 3, we discuss the role of yoga in management by a study of the literature. Based on these we also develop the hypothesis for our study. We present the study details in Section 4, and discuss the results in the following Section. Finally in Section 6, we conclude by highlighting the implications of our study.

## MATERIALS AND METHODS

### Factors contributing to organizational performance

Organizational performance can be termed as the achievement of the goals of an organization. The goals of an organization may differ from organization to organization and may also include in its fold quantitative and qualitative aspects. When an organization achieves its goals, it is said to have performed well. As performance is the main reason for the survival of an organization, there is considerable interest among practitioners and researchers to understand what results in a better organizational performance.

Marmol and Murray[3] studied High Performing Organizations (HPOs) from a variety of sectors including financial services, technology, consumer goods, retail manufacturing, transportation, customer services, and energy. The focus of the study was on identifying qualities and practices that helped organizations sustain a superior performance over a long period. They observed that of the six attributes that were common to the HPOs, the most important was leadership competence. According to Uma,[4] most organizations are impacted by globalization, new challenges, and complexities irrespective of whether they operate globally or not. Leadership competence is critical to the success of the organization perhaps more than ever before.

Prahalad,[5] discussed the challenges that leaders face in the current environment and the qualities required. The main competencies that he emphasized to face these are:

- Coping with ambiguities and uncertainties
- econciling the coexistence of opposites
- Managing the diversity in terms of race, age, gender, culture, and intellectual person integrity
- Selflessness
- Humility and courage
- Networking across organizations
- Contextual influence and authority

According to Tichy,[6] the single most important factor that differentiates winning companies from losing ones is that the winning companies possess a leadership engine — a proven system for creating dynamic leaders at every level. Warren[7] observed that the key to future competitive advantage will be the organization’s capacity to create a social architecture capable of generating intellectual capital; and leadership is the key to realize full intellectual capital.

The quality and disposition of managerial level employees is thus a key to organizational performance. Some of the factors that can measure these qualities are job satisfaction, job involvement, goal orientation, organizational commitment, and organizational citizenship behavior. We present a review of their influence on the quality of leadership and organizational performance.

#### Job satisfaction

According to Bullock,[8] job satisfaction is an attitude that results from a balancing and summation of many specific likes and dislikes experienced in connection with the job. Smith[9] has defined job satisfaction as an employee’s judgment of how well his job has satisfied his various needs. Blum and Naylor[10] have defined job satisfaction as a general attitude formed as a result of specific job factors, individual characteristics, and relationships outside the job. Robbins[11] too has defined job satisfaction as an employee’s general attitude toward his job.

In the mid-seventies, Locke[12] reviewed the research work done on job satisfaction during the preceding 40 years, beginning with the classic study by Hoppock.[13] Locke reported that more than 3000 studies had been published during the said period of 40 years. A critical review of the researches indicated that although there was no direct or consistent relationship between job satisfaction and productivity, the scholars and management practitioners were still interested in the study of job satisfaction for the following reasons, which had broad implications for the individual, the organization, and the society at large;

- Absenteeism is higher among dissatisfied employees[14,15]
- Dissatisfied employees are more likely to quit[16]
- Satisfied employees enjoy better health and live longer[12,17]
- Job satisfaction is infectious and carries over to life outside the work place[18]

In a survey of 440 commercial bank employees in Bangladesh, Mosharraf[19] concluded that job satisfaction had a significant positive contribution to performance. Judge and Bono[20] found through empirical evidence that self-esteem, generalized self-efficacy, internal locus of control, and emotional stability are among the best dispositional predictors of job satisfaction and job performance. Lopez[21] found that self-esteem moderates the job performance – job satisfaction relationship. Cropazano Bonnet (2007) established that the employees’ psychological well-being and employee morale have a moderating effect on the relationship between job-performance and job-satisfaction. Based on Korman’s Consistency Theory of Work Motivation, Inkson[22] established that self-esteem exercised a significant moderating effect on the correlation between performance and intrinsic satisfaction, but not on the correlation between performance and extrinsic satisfaction.

These studies point to the role ‘job satisfaction’ plays in creating a positive ambience for the employees, motivating them and thereby ensuring high productivity. These in turn are likely to contribute to the performance of an organization.

#### Job involvement

Job involvement is an important factor in the lives of most people. Work activities consume a large proportion of time and constitute a fundamentally important aspect of life for most people. People may be stimulated by and drawn deeply into their work or alienated from it mentally and emotionally. The quality of one’s entire life experience can be greatly affected by one’s degree of involvement in or alienation from work.[23,24] A state of involvement implies a positive and relatively complete state of engagement of the core aspects of the self in the job, whereas, a state of alienation implies a loss of individuality and separation of the self from the work environment. For example, Argyris,[23] Kanungo,[25] Marx,[26] McGregor,[27] Kanungo[25,28] considered involvement and alienation to be polar opposites.

Lawler and Hall[29] defined job involvement as a ‘psychological identification with one’s work’ and ‘the degree to which the job situation is central to the person and his (or her) identity’ (p. 310-311). Increasing job involvement can enhance organizational effectiveness and productivity by engaging employees more completely in their work, and making the work a more meaningful and fulfilling experience.[30]

The ‘individual difference perspective’ holds that job involvement results from socialization processes that inculcate the importance of work as a virtuous and necessary activity, as well as from other stable individual differences. This research draws on the work of Weber,[31] with its emphasis on individuality and the virtue of work as an end in itself. Such beliefs are likely to predispose people to be more job involved.[32–34] Individuals with an internal locus of control (i.e., those who believe they are active causal agents) are likely to be more job involved than individuals with an external locus of control.

Previous research has not established the causal ordering of job involvement with respect to job satisfaction and organizational commitment. We can classify job satisfaction as a consequence of job involvement, even though reciprocal causation is likely. One can consider job satisfaction primarily as a consequence, because cognitive appraisal of the potential for need satisfaction logically precedes actual need satisfaction. It is also likely that actual satisfaction then reciprocally influences job involvement. Conclusively, disentangling the causal priority of these two constructs empirically is likely to be difficult. Stumpf[35] concluded that both work performance and work satisfaction had antecedent influences on job involvement. All of these studies were co-relational, and none conclusively ruled out alternate causal orderings.

#### Organizational commitment

Robbins[36] has pointed out that dedicated or committed employees serve as ‘pivotal variables without which the inanimate assets are worthless’. Several research and consulting organizations[37] also suggest that a committed workforce is the ‘hallmark’ of a successful organization. ‘Committed or dedicated employees are expected to be more productive and work with focus on quality, to increase customer satisfaction and profitability of their organization’.[38] In a study of skilled workers of a private manufacturing unit (*n*=200) Pal,[39] found that a humane and fair management style significantly related to organizational commitment. Objectivity and rationality was found to be significantly related to organizational commitment in a study undertaken by Sharma.[40]

In a study conducted on 400 employees at the Indian Institute of Management Bangalore, Adhia[41] found that three factors, organizational politics, distributive justice, and procedural justice are strong predictors of affective organizational commitment. In the regression of affective organizational commitment, taking these three as predictors, the adjusted R square comes to 0.224, with *P*<0.01.

It appears from this that organizational commitment is an obvious contributor to organizational performance because loyalty to the organization significantly enables the organization to achieve its objectives. The primary difference between organizational commitment and job involvement is that job involvement primarily reflects one’s attitude toward a specific job, whereas, organizational commitment refers to one’s attachment to the organization.[42,43] It would be possible, for example, to be very involved in a specific job but not be committed to the organization and vice versa.[44,45]

#### Organizational citizenship behavior

Organizational Citizenship Behavior (OCB) pertains to the employees’ behavior over and above the call of duty (job description and job specification), which is very important for organizational effectiveness. The globalization of the industrial world makes it imperative for organizations to put special emphasis on organizational innovation, flexibility, productivity, and responsiveness to changing external conditions for their performance. It has been increasingly felt that work behavior such as OCB, which is beyond the reach of traditional measures of job performance, holds promise for long-term organizational performance. A comprehensive theoretical discussion is available in the works of Organ,[46] Konovsky and Pugh,[47] Moorman and Blakey,[48] and Padsakoff and MacKenzie.[49] Attempts are also made to assess the probable factors (causes) which may lead employees to foster organizational citizenship behavior.[50–54]

In 1983, Denis Organ and his colleagues were the first to use the term OCB.[50,51] Later, drawing on the concept of ‘willingness to cooperate’ based on Bernard’s,[55] the distinction between dependable role performance and innovative and spontaneous behaviors, Organ, defined OCB as an individual behavior that was discretionary, and not directly or explicitly recognized by the formal reward system, and that in aggregate promotes the effective performance of the organization.

This concept has also been characterized as including constructive and cooperative extra role gestures that are neither mandatory nor directly compensated by a formal organizational reward system. In addition such behaviors have been described as having an accumulative positive effect on organizational functioning. Bateman and Organ[51] attempted to cluster a list of employee behaviors that managers typically need and appreciate, but are helpless to demand. These behaviors also formed part of what they called OCB. Included in the list are gestures such as, constructive statements for improvement of the organization/ department, expressing personal interest in the work of others, monitoring the new entrants in the organization, respecting the spirit as well as the rules of the organization, care for organizational property, and so on. It also takes into account specific behaviors that employees refrain from indulging in, even though they may have every right to do so. To be more specific, these behaviors include finding fault with coworkers/ managers, expressing resentment, complaining against trivial/ insignificant issues, arguing with others, and so forth. The contention behind including such behaviors within the purview of the concept is that OCB does not only include enactment of positive gestures and contributions, but it also takes into account the quality of forbearance.

Clearly, the concept of OCB induces behavioral and attitudinal patterns on the part of managers that influence organizational performance.

#### Goal orientation

Goal orientation refers to taking one’s goals seriously and being persistent in pursuing the goal. Achievement goal theory and research suggest that employee job performance and job satisfaction depend on their goal orientations.[56,57] Goal orientation can be regarded as a personality concept, implying the existence of individual differences in the extent to which people set goals and pursue them. Highly goal-oriented persons develop long range and clear goals. They are persistent in pursuing them, especially when difficulties arise. Therefore, goal orientation is assumed to be an important prerequisite for effective leadership.

Previous research has shown that a person’s goal orientation was related to his or her performance in individual settings.[58] In a study reported by Sonnentag, Stolte, Frese, Heinbokel, and Brodbeck,[59] it was stated that the team leaders’ goal orientation is related to the quality of the development process, the quality of the final product, and the interaction within the team. The goal orientation of individuals in an organization does lead to focused action, which helps in achieving organizational objectives.

On the basis of the review of literature one can make certain inferences pertaining to factors influencing organizational performance. We summarize them below:

- The quality and disposition of managerial level employees is the key to organizational performance.
- These are indeed reflected in some organizational indicators, such as, job satisfaction, job involvement, goal orientation, organizational commitment, and organizational citizenship behavior.
- These factors are suitable for our study also, because they can be studied and measured in respect of any organization, irrespective of their goals or line of business.

#### Yoga way of life

The yoga way of life encompasses the philosophy of *Karma Yoga* (path of detached action), *Jnana Yoga* (knowledge of self), *Bhakti Yoga* (Trust in the supreme order) and *Raja Yoga* (*Asana, Pranayam*, Meditation etc.). Practicing this knowledge may bring about a complete transformation of one’s personality, on the physical, mental, emotional, and spiritual levels, which strengthens one’s ability and desire to put in one’s best. Yoga is one of the six foundations of Indian philosophy and has been used for millennia to study, explain, and experience the complexities of the mind and human existence.[60] *Patanjali*, an ancient yoga sage, defines yoga as a technique used to still the mental fluctuations of the mind to reach the central reality of the true self.[61] *Patanjali’s Yoga Sutras* outline a skillful way of conducting life that fosters moderation and harmony.[62] These guidelines, which include ethical and moral standards of living in addition to postural and breathing exercises, are used to foster spiritual growth and evolve one’s consciousness.

*Ashtanga Yoga*, the eight step path of yoga, encompasses cognitive learning, moral conduct, physiological practices, and psychological therapy. The first two steps of *Yama* and *Niyama* seek and shape external behavior and thought patterns and thus minimize disturbances in the mind and the body. On the behavioral side, abstinence is sought from violence, falsehood, dishonesty, sexual excess, and acquisitive tendencies. On the cognitive moral side, the ideals prescribed are — purity, contentment, austerity, self study, and forbearance. The stages of *Asana* and *Pranayama* are meant for disciplining the body and regulating subtle energy flows. In the fifth stage of *Prayahara*, the secondary input is regulated so the mind is not distracted. The stages of *dharana, dhyana*, and *Samadhi* are for uplifting one’s spiritual self and for heightening consciousness.

According to Srinivas,[63] a series of techniques collectively known under the general label ‘Yoga’ present a rich source for generating indigenous organizational development techniques that may perhaps find better acceptance than imported intervention designs from the west (p. 271). Originally developed for personal spiritual growth, yoga offers a well-formulated approach to planned change.[63]

#### Impact of yoga on management

Spirituality in a workplace is a topic of hot discussion today. Wisnieski and Askar and Syed[64] present four interesting advantages in their review of workers who maintain a spiritual mindset. First they claim: ‘The stronger the spiritual factor of the personality, the more tolerant the person is of work failure and less susceptible to stress’ (p. 102). Second, these authors assert, ‘the stronger the spiritual factor of the personality the more the person favors the democratic style of leadership, is more trusting and the higher is his/ her tolerance of human diversity’. Third, it is the opinion of Mohamed *et al*. that, ‘The stronger the spiritual factor of the personality the more the person exhibits altruistic and citizenship behavior’. Finally, these authors find that, ‘The stronger the spiritual factor of the personality, the more the person’s commitment to the organization and work group increases’.

In the article of Sangster,[65] he re-emphasizes an often presented clarification when the topic of spirituality in the workplace is mentioned, he places religion out of the scope, stressing that ‘it is possible to lead a spiritual way of life without following any particular religious path’ (p. 16). In Sangster’s opinion, spiritual workers are those who think cooperatively and/ or altruistically; have a balanced, objective view of the world; listen as much as (or more than) they speak; apply a three-dimensional or bigger picture when thinking; believe in some higher driving force and purpose beyond humankind; find the time to think things through objectively; think laterally in order to promote realistic solutions; encourage and empower others selflessly; work open-mindedly with a wide range of people; consistently display integrity and trust; and expect the best from people without having a soft touch. (p. 16)

Jurkiewicz and Giacalone[66] stress yet another major advantage of nurturing the spiritual mindset within each worker in the organization: ethicality. These authors assert that the ‘Fundamental aspects of workplace spirituality, such as meaningful work that provides a feeling of purpose, a sense of connection and positive social relations with their coworkers, and the ability to live an integrated life in which the work role does not conflict with the essential nature of the person as a human being, may interact to create different perceptions of ethicality within the organization’ (p. 85). Most of the work available on the subject of the impact of yoga on work life/ management centers around the impact of Transcendental Meditation on various aspects of management. A review[67] of over 500 experimental studies conducted in over 200 universities, in 33 countries, revealed that Transcendental Meditation helps expand consciousness, decrease oxygen intake and stress level, increase basal skin resistance and coherence in EEG, and virtually suspends breathing up to one minute.

Transcendental Meditation is a skill of effortlessly minimizing mental activity so the body settles into a state of rest deeper than deep sleep, while the mind becomes clear and alert. At the University of Texas, Orme-Johnson *et al*.[68] showed that mediators display a greater physiological equilibrium than non-mediators. He also showed that mediators maintain this equilibrium under stress more effectively than non-mediators. David[69] completed a study, which concludes that TM increases individual productivity. David found that mediators show increased job satisfaction, a decreased desire to change jobs, better performance, and better relationships with supervisors and co-workers. Findings on the TM technique relevant to organizational performance include, improved cognitive performance,[70] increased self-esteem,[71] and higher levels of self-actualization and development,[72,73] associated with more effective managerial performance.[74] Previous case studies suggest that large proportions of organization members practicing the Transcendental Meditation technique have contributed to improvements in organizational performance.[75]

Pande and Naidu[76] reported empirical evidence to show that people having a strong orientation to *niskam karma* (working sincerely without being preoccupied with the outcome) experience less work-related stress. Misra[77] found that effort orientation rather than concern for the outcome leads to greater intrinsic satisfaction. Chakraborty[78,79] provides experimental evidence that practicing yoga, meditating, controlling breathing, and stilling the turbulent mind can enable workers and managers to purify their chitta and make it spiritual, expand their self to include others around them, and help them grow and transform themselves, without expecting anything in return.

From a complete review of the literature, one finds that there have been attempts in the past research to capture the positive impact of some aspect of yoga (such as asana or meditation) on personal factors such as stress, and so on. However, there has been no attempt to capture the effect of the adoption of complete yoga philosophy on organizational factors. As we have seen in the literature review, Yoga has a deeper psychological impact on a person, in terms of changing paradigms of one’s life. Similarly most of the organizational factors selected for this study are psycho-motivational in nature. There is, therefore, enough ground for us to hypothesize that adoption of yoga as a philosophy and practice helps in increasing the levels of positive organizational factors. This research is an attempt to prove or disprove this hypothesis.

### Empirical study details and results

This controlled experiment was conducted at a manufacturing company in Gujarat, called Birla Celluloise, located at Kosamba, one of the units manufacturing Viscose Staple Fiber, owned by the Grasim Industry, located at Kharach village near Bharuch. This unit has more than 120 people in the managerial cadre and more than 1000 in the workers category. Most of them stay in the township of the company, which makes it easy to conduct the intervention of Yoga. Grasim Industries Limited, a flagship company of the Aditya Birla Group, ranks among India’s largest private sector companies, with consolidated net revenues of Rs. 141 billion and a consolidated net profit of Rs. 20 billion (FY2007). Starting as a textiles manufacturer in 1948, today Grasim’s businesses comprise viscose staple fiber (VSF), cement, sponge iron, chemicals, and textiles. Its core businesses are VSF and cement, which contribute to over 90 percent of its revenues and operating profits. The Aditya Birla Group is the world’s largest producer of VSF, commanding a 21 percent global market share. The company meets India’s entire domestic VSF requirements. Grasim’s VSF plants are located at Nagda in Madhya Pradesh, Kharach in Gujarat, and Harihar in Karnataka, with an aggregate capacity of 270,100 tons per annum (tpa). The VSF plant at Kharach, where this experiment was conducted, was set up in 1996.

**Figure 1:**
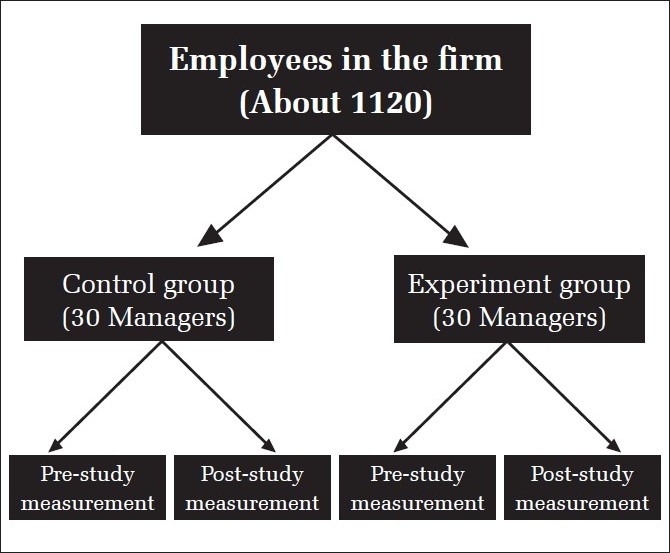
A schematic representation of the study plan

The salient aspects of the study methodology are summarized below:

- The managers of the company were given the option of joining this experiment after explaining to them the purpose and modality of this experiment and making clear to them the expectations of regularity and so on. Written consent for being a part of the experiment was obtained.
- Those who opted were initially divided in two equal groups of 42 each — Group one was called the Yoga group and Group 2 was called the physical exercise group, which was the control group for this experiment.
- The Yoga group was given 30 hours of yoga practice (75 minutes every day) and 25 hours of theory lectures on the philosophy of yoga. The total intervention period was six weeks. The theory lectures were given by the first author of this article and included topics such as the definition of the yoga way of life, implications of the four types of yoga (*Raja Yoga, Karma Yoga, Jnana Yoga* and *Bhakti Yoga*) on life, analysis on the aspects of true happiness in life, *ashtanga yoga* steps, central theme of universality of consciousness as given in *Vedanta*, and so on. The practice was given for *asanas, pranayama, Kriya*, and relaxation by a well-trained yoga instructor.
- The control group was also given training of equal number of hours for normal physical work-out and lectures on success factors in life (based on modern thought). This was thought necessary in order to obviate the possibility of the Hawthorne effect on the experiment group. The topics for theory given to this group included — Success and Happiness, Importance of Attitude, Self-Image, Good Relationship with Others, Goal Setting, Power of sub-conscious mind, Communication, Motivation, and leadership. The practice given to this group was fast exercises such as spot jogging, bending, body rotation, hand and leg movements, and the like.
- In order to prove or disprove the hypotheses, variables were measured for both the groups, before and after the experiment, with the help of a standard self-reported questionnaire. In addition, the measurement of certain physical parameters such as weight, BMI, BP, Blood Sugar, and so on, were taken for all, both pre- and post-experiment.
- The pre-measurement data was taken on 17 September, 2007. The intervention to both the groups was simultaneously given between the 18 September and 24 October, 2007. The post-measurement data was taken on 24 October, 2007.
- Out of the group of 42 in both groups, there were some who did not attend all theory and practice classes on many days, and hence only top 30 (in terms of regularity) were included for both groups in the final sample, for the analysis. This came to a minimum attendance figure of 65% approximately for both groups. In short, people with less than 65% aggregate attendance were excluded from both groups. The profile of the sample finally included in the experiment can be seen in Tables 1 and 2. Figure 1 schematically shows the study methodology and the group composition.

**Table 1:** Profile of sample-age wise age2* group crosstabulation

				Group		
				1 Yoga	2 Physical	Total
Age 2	1.00	21-50	Count	24	26	50
			% within group	80.0	86.7	83.3
	2.00	51 and above	Count	6	4	10
			% wthin group	20.0	13.3	16.7
Total			Count	30	30	60
			% within group	100.0	100.0	100.0

* Table interpretation: The age profile of yoga group and the control group is almost similar

**Table 2:** Profile of sample-level of management group crosstabulation

			Group*		Total
			1 Yoga	2 Physical	
Level of management	I line level	Count	17	19	36
		% within group	56.7	63.3	60.0
	M middle level	Count	7	6	13
		% within group	23.3	20.0	21.7%
	T top level	Count	6	5	11
		% withn group	20	16.7	18.3
Total		Count	30	30	60
		% within group	100.0	100.0	100.0

* Table interpretation: The level-wise division of both the groups is almost homogenous

#### Constructs used for measurement of variables

Questionnaires were both easy to administer and inexpensive,[80] due to their brevity and self-reportive nature. It was therefore decided to use self-reported questionnaire, pre- and post-intervention, for this study. Intrinsic job satisfaction was measured using the shorter version of the Minnesota Job Satisfaction Questionnaire, popularly known as MSQ,[81] from which items loading highly on the ‘intrinsic’ factor were chosen.[48] These items were related to the opportunity the respondent had to make use of, his/her skills and abilities, the trying of new ideas and methods, and the feeling of accomplishment that was generated on the job. This scale was preferred over other scales such as Job Descriptive Index,[82] because they were lengthy and multidimensional. Furthermore, the MSQ was the most cognitive in its orientation.

Affective Organizational Commitment was measured using eight items pertaining to the affective component of organizational commitment, from the instrument developed by Allen and Meyer,[83,84] which is responded to on a seven-point Likert type scale. The items are used to tap the extent of the employee’s emotional attachment to, identification with, and involvement in the organization. Job Involvement was measured using the 13-item job involvement-role scale developed by Paullay, *et al*.,[85] with responses taken on a seven-point Likert type scale. It was used to tap the extent to which the respondent was involved personally in the type of work that he/she did in his/her present job.

Organizational Citizenship Behavior (OCB) was measured using a slightly modified version of the scale developed by Moorman and Blakely,[48] based on Graham’s[86] four-dimensional model of OCB. The modification was mainly intended to facilitate self-reporting. The four dimensions proposed by Graham were Interpersonal Helping (IH), Personal Initiative (PI), Loyal Boosterism, and Personal Industry. However, only items related to Interpersonal Helping and Personal Initiative were included in the scale, because of the potential for a high level of social desirability of other items.

Goal orientation was measured with a scale developed by Frese, *et al*. It is argued that while measuring goal orientation, it is optimal to refer to the same situations for all respondents, due to project difference. However, Frese, *et al*. have showed that there is no need to provide a situational input for the goal, as there is a certain degree of cross-situational generality in the goal orientation scale. The four items used to measure goal orientation were anchored on a seven-point scale with 1 standing for ‘strongly disagree’ and 7 standing for ‘strongly agree’.

The data collected was analyzed using SPSS. The sample profile given in Tables 1 and 2 indicated that 80 and 86% of the participants from the yoga and control groups, respectively, were from the age group of 21 to 50, while the rest were above 50. Similarly, 20% of the yoga group and 17% of the control group were from the top management (i.e., Deputy General Manager and above), while 57% of the yoga group and 63% of the control group were from the line-level managers (Deputy Managers and officers). The average total work experience of the sample group was 16.11 years.

Table 3 shows that at Baseline there is no difference in any indicator (Independent Sample *t* test). It can be concluded from Tables 4 and 5 that in the yoga group, remarkable positive changes are observed in all the Indicators except Job Involvement (Paired *t* test), unlike in the physical exercise group where the post intervention impact on indicators do not show a statistically significant difference in any of the five indicators. Table 6 shows that although, there is significant improvement within the Yoga group in four of the indicators (except JI), the comparison of Post Intervention Averages between the two groups using t-test exhibits that the two groups after intervention (at endpoint) do not differ significantly in any one of the five variables. The changes that are occurring in other indictors remain small in terms of effect. One reason for this may be the limited time frame of the study (two months). Furthermore, in many cases the initial level of all five factors in this organization was already high for both the groups. It appears that in the long run the Yoga group may turn out to be improving significantly in all indicators compared to the physical group.

**Table 3:** Comparison of indicators at baseline between yoga and physical exercise groups

Group	N	Mean	Std. deviation	Significance of independent samples *t*-test
2 physical exercise	30	5.6131	.56774	
Pre AOC				
1 yoga	30	4.5667	1.21047	.067
2 physical exercise	30	5.0875	.93319	
Pre JI	30	5.1442	.73501	.233
1 yoga				
2 physical exercise	30	5.3452	.54101	
Pre GO	30	4.8000	1.23770	.146
1 yoga				
2 physical exercise	30	5.2917	1.34722	
Pre OCB	30	5.4033	.93973	.082
1 yoga				
2 physical exercise	30	5.7800	.68752	
Pre JS	30	4.9677	.98603	.234
1 yoga				
2 physical exercise	30	5.2630	.91554	

Table interpretation: It is seen from the significance column in the above table that at Baseline there is no difference in the mean score of any indicator between the two groups at baseline

**Table 4:** Post intervention paired comparison to see improvement/deterioration in each indicator separately for yoga and physical exercise groups

Yoga group	Mean	N	Std. deviation	Mean difference	*t*	Sig. (2-tailed)
Pre AOC	4.5667	30	1.21047	-.5911	-2.239	.033
Post AOC	5.1577	30	1.19700			
Pre JI	5.1442	30	.73501	-.2301	-1.335	.192
Post JI	5.3744	30	.55607			
Pre GO	4.8000	30	1.23770	-1.1417	-4.672	.000
Post GO	5.9417	30	.81654			
Pre OCB	5.4033	30	.93973	-.7052	-2.784	.009
Post OCB	6.1085	30	.83496			
Pre JS	4.9677	30	.98603	-.7693	-3.477	.002
Post JS	5.7370	30	.76047			

Table Interpretation: For the yoga group the change in the post-intervention score over the pre-intervention score is significant for all the Indicators except JI. Significant improvement is observed in AOC (Increase), GO (Increase), OCB (Increase), JS (Increase)

**Table 5:** Physical exercise group

	Mean	N	Std. deviation	Mean difference	*t*	Sig. (2-tailed)
Pre AOC	5.0875	30	.93319	.3792	1.621	.116
Post AOC	4.7083	30	1.02361			
Pre JI	5.3452	30	.54101	-.0600	-.336	.739
Post JI	5.4051	30	.78653			
Pre GO	5.2917	30	1.34722	-.4667	-1.623	.116
Post GO	5.7583	30	.77538			
Pre OCB	5.7800	30	.68752	-.0567	-.329	.744
Post OCB	5.8367	30	.55739			
Pre JS	5.2630	30	.91554	-.2481	-1.364	.183
Post JS	5.5111	30	.65676			

Table interpretation: For the yoga group the change in the post-intervention score over the pre-intervention score is significant for all the indicators except JI. Significant improvement is observed in AOC (Increase), GO (Increase), OCB (Increase), JS (Increase)

**Table 6:** Post-intervention mean scores comparison between two groups

Group	N	Mean	Std. deviation	Significance of independent samples *t*-test
Average post AOC				
1 yoga	30	5.1577	1.19700	.124
2 physical exercise	30	4.7083	1.02361	
Average post JI				
1 yoga	30	5.3744	.55607	.862
2 physical exercise	30	5.4051	.78653	
Average post GO				
1 yoga	30	5.9417	.81654	.376
2 physical exercise	30	5.7583	.77538	
Average post OCB				
1 yoga	30	6.1085	.83496	.143
2 physical exercise	30	5.8367	.55739	
Average post JS				
1 yoga	30	5.7370	.76047	.223
2 physical exercise	30	5.5111	.65676	

Table interpretation: There is no significant difference between the two groups post the experiment

Table 7 shows the Pearson’s correlations, post-experiment, among the five variables measured. The results show that most of these five variables are strongly correlated, which means the presence of one factor will mean the presence of other factors also. Only job involvement does not show significant correlation with job satisfaction. Interestingly, the construct used for measuring job involvement had a Chronbach alpha of 0.613, which is an indicator of moderate reliability.

**Table 7:** Correlations

	Average post AOC	Average post JI	Average post GO	Average post OCB	Average post JS
Pearson correlation	1	.334(**)	.367(**)	.373(**)	.349(**)
Sig. (2-tailed)		.009	.004	.003	.006
N	60	60	60	60	60
Pearson correlation	.334(**)	1	.335(**)	.422(**)	.248
Sig. (2-tailed)	.009		.009	.001	.056
N	60	60	60	60	60
Pearson correlation	.367(**)	.335(**)	1	.452(**)	.536(**)
Sig. (2-tailed)	.004	.009		.000	.000
N	60	60	60	60	60
Pearson correlation	.373(**)	.422(**)	.452(**)	1	.517(**)
Sig. (2-tailed)	.003	.001	.000		.000
N	60	60	60	60	60
Pearson correlation	.349(**)	.248	.536(**)	.517(**)	1
Sig. (2-tailed)	.006	.056	.000	.000	
N	60	60	60	60	60

** Correlation is significant at the 0.01 level (2-tailed)

### Yoga as a viable and positive organizational tool

Today, there is considerable interest among the management practitioners and researchers on the role and benefits of introducing spirituality at the workplace. The Harvard Business School study drawn over a period of 11 years showed a marked relation between the strength of the organizations’ corporate culture and its profitability.[1] Lloyd maintains that organizations high in workplace spirituality outperform those without it by 86%.

According to Sharma,[87,88] Indian management ideas such as yoga in management, Vedanta in management, and the Kosha model in management offer new approaches to the concept of management, wherein competitive advantage, collective advantage, and *karma* advantage are balanced. According to Sharma, the Indian word ‘Udyoga’ (which means Industry) contains in itself the word ‘yoga’. Thus, in Indian management thought, Udyoga is a yoga (*Udyog hi yog hai/Udyog bhi ek yog hai*). In popular terms Sharma[87,88] refers to this as the BHMS (Body–Heart–Mind–Soul) model. There is an interactive relationship between the Body, Heart, Mind, and Soul. They influence each other in varying ways. Even organizations can be conceptualized as BHMS systems. With the arrival of the services and knowledge economy, the need for the BHMS approach to management is getting attention. This implies a shift from a fragmented view to a holistic view of human beings, society, and organizations.

Vedanta in management implies management by higher values and higher vision. Thus, YVK (Yoga, Vedanta, Kosha) constitute three ‘Eastern Doors’ that can be combined with ‘Western Windows’ (traditional Western Management theories and concepts). These ideas can be combined with various wisdom traditions to arrive at a new philosophy of what Sharma[87] calls ‘Western Windows, Eastern Doors, and Wisdom Corridors’ in the field of management and related social sciences.

How can we explain the result, obtained in this experiment, is a moot question? Yoga Way of Life, the concept presented in this article is an integrated approach of the changing physical, mental, vital, and emotional personality of an individual. It aims at making managers more evolved individuals, with a better understanding of their job situation in the overall context of life. The teachings of *Karma* Yoga are useful in changing outcome orientation to effort orientation, and in reducing the managers’ expectations from the job. The system of Yoga is analytical and makes an individual more aware of his situation and allows him to give a considered rather than an intuitive response to it.

Yoga psychology conceives the self (*atman*) in terms of different levels of being. The inner-most core or *atman* is covered by hierarchy of five sheaths or layers. This continuation of layers corresponds to a sort of stepwise ladder, leading inward to the *atman*; the journey inward forms the basis of growth and development: Biological evolution from a protozoan to man, psychological evolution from child to adult, consciousness enhancement from mere cognitive to universal consciousness, wherein there is no ego and there is realization that the concerns and needs of all people are the same, and that what is good for one is good for all. In this growth process, feeling and emotions are accepted as having a place, they are not considered wrong or repressed, but are transformed and redirected.[63]

According to Rama Swami *et al*.,[89] Yoga psychology integrates behavioral and introspective approaches to growth. It provides a perspective by which one can become disengaged from involvement in the unhappy personalities he has created for himself and in the negative role he has adopted. It moves quickly to a training program for changing habits, thought patterns, and self concepts.

The positive results obtained in this research confirm this. Yoga Philosophy helps a person to have a broader view of life, with greater awareness of his actions. This automatically results in enhanced commitment to his job and organization. It also means that his willingness to go beyond the call of his duty is a result of his understanding the concept of *karma* yoga, in which the performer does not depend on rewards for his/ her performance. The high level of OCB found in the Yoga group here can be attributed to this. Furthermore, as at least four out of the five factors measured here are strongly correlated, this positive impact is also found in job satisfaction, affective organizational commitment, and so on.

Some people may be unnecessarily apprehensive about the renunciation effect that introduction of this philosophy may have on the drive or killer instinct of their executives. Such apprehensions come out of the wrong understanding of the true concepts of yoga. For example, far from being against ‘goal orientation’, the concept of *Karma* Yoga is so dynamic in nature that it frees an individual from all worries and propels him to action immediately. Also a person who is not excessively worried about the results can only be a true risk-taker, who will take tough decisions in the best interest of his organization. The results of this research show that the yoga way of life can bring about better ‘Goal-orientation’ among managers.

In most organizations, leaders play a pivotal role in driving performance. There are varieties of leadership training programs being tried nowadays by successful companies. However, the yoga way of life is rarely taught in these training programs. At the most, yogasanas are being taught to the group, as part of a morning physical work-out. There are few companies who have of late started providing meditation rooms at the work place, for the ease of managers who want to go into solitude to sharpen their creativity.

It may be a good idea to now start thinking of giving systematic exposure of wisdom, enshrined in our ancient scriptures, to all managerial cadres of companies, which would help them personally as well as professionally. They can become better self-aware and self-regulated individuals, with a proper perspective of life and various relationships. The Yoga way of life is all about the correct attitude to life, which can result in a better organizational climate. In the Indian context, the assimilation of this knowledge may be better and easier, as the Indians have grown with all these concepts right from childhood.

’How can one proceed in this?’ is a very important question. First of all, the top leaders of the company have to be convinced about the utility of this idea. They should themselves have the necessary trust in this philosophy and in the results it can bring. Once convinced about the utility of this kind of training, the tougher challenge lies in finding the right people to train company executives. And second, this has to be a continuous training, which is repeated periodically. Third, the atmosphere of the yoga way of life has to be created in the company policy. The company policies have also to pass the test of the yoga way in terms of completely adhering to the ethical-moral code prescribed in Yama and Niyama.

## CONCLUSION

There are certain organizational factors, such as Job Satisfaction, which have a crucial bearing on organizational effectiveness. Our study confirms the useful role that the Yoga way of life can play in improving these factors positively among managers. The results show a significant difference among those who are introduced to the practice of the yoga way of life. However, in order to get the benefit of yoga in its entirety, one has to adopt yoga as a technique of life management. This would include not only the Raj Yoga practices of *asana, pranayama*, and meditation, but also imbibe the concept of detatched action (*Karma Yoga*), trust in God’s justice system (Bhakti Yoga), and seeking the knowledge of self (Jnan Yoga). Such an integrated approach can yield superior results for individual happiness and also for organizational performance.

On the basis of our study we are motivated to recommend similar experimental studies in multiple organizational settings to further refine these findings and insights. One approach is to conduct a similar study with a large sample size, which may corroborate this initial attempt. Also in case of other similar experiments, different instruments for measuring these factors may be tried, in order to take care of social desirability.
